# A rapid method of fruit cell isolation for cell size and shape measurements

**DOI:** 10.1186/1746-4811-5-5

**Published:** 2009-04-29

**Authors:** Peter A McAtee, Ian C Hallett, Jason W Johnston, Robert J Schaffer

**Affiliations:** 1 The New Zealand Institute for Plant & Food Research, Private Bag 92169, Auckland 1142, New Zealand

## Abstract

**Background:**

Cell size is a structural component of fleshy fruit, contributing to important traits such as fruit size and texture. There are currently a number of methods for measuring cell size; most rely either on tissue sectioning or digestion of the tissue with cell wall degrading enzymes or chemicals to release single cells. Neither of these approaches is ideal for assaying large fruit numbers as both require a considerable time to prepare the tissue, with current methods of cell wall digestions taking 24 to 48 hours. Additionally, sectioning can lead to a measurement of a plane that does not represent the widest point of the cell.

**Results:**

To develop a more rapid way of measuring fruit cell size we have developed a protocol that solubilises pectin in the middle lamella of the plant cell wall releasing single cells into a buffered solution. Gently boiling small fruit samples in a 0.05 M Na_2_CO_3 _solution, osmotically balanced with 0.3 M mannitol, produced good cell separation with little cellular damage in less than 30 minutes. The advantage of combining a chemical treatment with boiling is that the cells are rapidly killed. This stopped cell shape changes that could potentially occur during separation. With this method both the rounded and angular cells of the apple cultivars SciRos 'Pacific Rose' and SciFresh 'Jazz'™ were observed in the separated cells. Using this technique, an in-depth analysis was performed measuring cell size from 5 different apple cultivars. Cell size was measured using the public domain ImageJ software. For each cultivar a minimum of 1000 cells were measured and it was found that each cultivar displayed a different distribution of cell size. Cell size within cultivars was similar and there was no correlation between flesh firmness and cell size. This protocol was tested on tissue from other fleshy fruit including tomato, rock melon and kiwifruit. It was found that good cell separation was achieved with flesh tissue from all these fruit types, showing a broad utility to this protocol.

**Conclusion:**

We have developed a method for isolating single cells from fleshy fruit that reduces the time needed for fruit cell separation. This method was used to demonstrate differences in cell size and shape for 5 different apple cultivars. While firmness between the different cultivars is independent of cell size, apples with more angular cells appear to be firmer.

## Background

Cell shape and size are important determinants of fruit size and texture. Recent reports investigating these links include *Solanum lycopersicum *(tomato) [1]*Malus × domestica *(apples) [2], *Prunus avium *(Sweet cherry) [3,4]*Diospyros species *(Persimmon) [5], *Prunus persica *(peach) [6] and *Musa species *(banana) [7]. In these cases, cell number or cell size was either estimated from fixed tissue that had then undergone sectioning, or cell maceration using chemical or enzymic digestions over 12 to 48 hours. However, the labour, chemical and time intensive nature of these techniques limits the number of samples and treatments that can analysed. There is increasing demand from fruit physiologists and breeders for more rapid techniques that allow analysis of larger numbers of fruits so that more robust conclusions can be derived about the importance of cell size and packing in fruit quality.

In apple fruit, texture is a primary consumer preference, making this a principle target for apple fruit breeders and pomologists. Texture is a complex trait, determined by the interaction of many factors such as cell wall chemistry, cell size and shape, cell packing and cell turgor [8]. Cell size has been shown to be one of the critical components for textural differences in apple, with juiciness being associated with larger cells [9,10]. Microscopy studies of bite action have shown that high levels of juiciness are achieved when cells are broken open, whereas when the fracture occurs between cells low levels of juiciness are found [11]. Apples have an extensive breeding history which has lead to the availability of many of cultivars displaying a wide range of fruit characters for size and texture.

Apple fruit flesh or cortex comprises of homogeneous parenchyma-type cells. There is a variation of cell sizes across the apple fruit with cells under the skin being smaller (70 uM), increasing in size (to approx 250 uM) towards the centre of the flesh [12,13]. Towards the inner cortex, the apple cells become more elongated, spreading out in a radial pattern, lying alongside air gaps [14]. Growth within the apple fruit varies according to position, with more rapid growth occurring at the calyx than at the stalk [15]. Growth in apple fruit is achieved through a combination of cell division and cell expansion and, unlike other fruit, enlargement of air gaps between cells [16,17]. Apple fruit continue to increase in size right up to harvest, albeit at a reduced rate once maturity has been reached [12,18]. Fruit size can be altered by crop load or environmental effects, and results from changes in both number and size of cells [19]. While there can be a considerable range of cell size within a cultivar, cell size is also genetically determined with apples like 'Bramley's Seedling' having particularly large cells [13,18,20].

One of the main issues with measuring cell size using fixing and sectioning is that often the cells are not spherical, and so the plane of sectioning will determine the size measurement. Additionally, there is no guarantee that you are viewing from the widest point of the cell. One solution to address this is to separate the individual cells of the tissue. The intercellular adhesion of plant cells is dependent on pectin, which is the major constituent of the middle lamella and, to a lesser extent, also found in plant cell walls, [21]. Pectinate polysaccharides are complex carbohydrates that consist of a backbone of 1,4 linked alpha-galacturonic acid subunits otherwise known as homogalacturonan, with occasionally 1,2 linked rhamnose subunits. The rhamnogalacturonan I facilitate the linkage of neutral sugar side chains, consisting of arabinans, galactans and arabinogalactans, which give pectin its adhesive properties by allowing pectin to bond with various other cell wall components. The strength of binding between the side chains of the pectic acid backbone is dependent on the presence of calcium and magnesium, which are involved in the cross-linking of pectic polymers. These cofactors fortify the adhesive properties of the pectic substances. Initially cell separation was achieved by treating the tissue with solutions containing chromic acid [22], or combinations of chromic acid and nitric acid [7]. This was later modified to use cell wall digesting enzymes [1,23,24]. The use of enzymes to separate cells has traditionally been associated with long incubation periods (12 to 48 hours) increasing the possibility of cell shape changes as the pectins are digested and/or changes in turgor.

In apples, cell size is not only determined genetically, but also by environment, crop load and maturity. Due to this complexity there is a need to assay a large number of apples to tease out the genetic component of size. We aimed to develop a more rapid, method to measure cell size using isolated cells. To separate the cells, we used Na_2_CO_3 _which is known to solubilise pectins [25]. We utilised this method to measure cell size and shape in different apple cultivars with known differences in texture to establish whether cultivar related differences could be observed using this technique. Finally we tested this method against other fleshy fruit to assess its utility as a more general cell isolation method.

## Results and discussion

### Isolation of single cells

**Figure 1:**
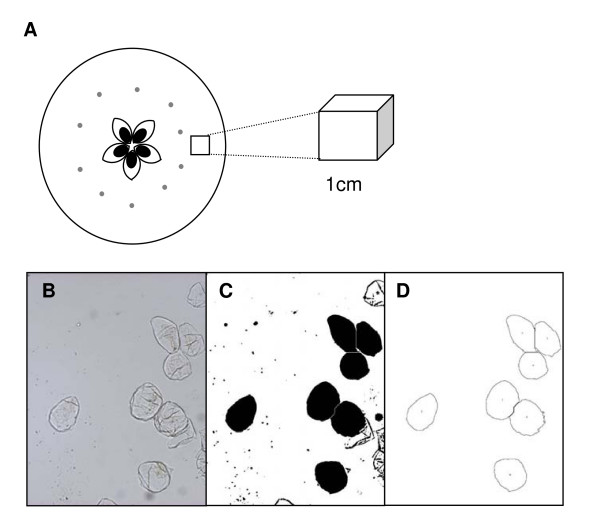
**Cell isolation and image analysis of the individual apple cells using the ImageJ software package**. Apple sampling location (A). Analysis steps of isolated cells (B-D), isolated cell images (B) are converted to binary and filled (C), after using a size threshold of 1100 pixels only the large intact cells are selected (D).

Cortex wedges from a 1 cm thick equatorial slice of apple (cv. 'Royal Gala') were cut with a paring knife. To avoid the small cells found near the skin, a block of approximately 1 cm^3 ^of cells from the central cortex were selected (figure 1A). To reduce the number of damaged cells from the initial cut approximately 0.1 cm of tissue was trimmed from each surface of each wedge using a fine edged scalpel blade. The resulting cortex tissue was then cubed into approximately 2 mm^3 ^tissue blocks with the fine edge scalpel. Single cell isolation was achieved in small 50 ml glass beakers using 40 ml of 0.05 M Na_2_CO_3 _in 0.3 M mannitol. Mannitol was used to osmotically buffer the apple cells in their physiologically normal osmotic range [11]. To delocalise gases present in the air gaps and to aid separation, the cubes were gently boiled on a magnetic hot-plate stirrer (180 rpm) for 20–30 minutes stirring with a 2 cm magnetic stirrer, the heat was then reduced until boiling stopped and the tissue was stirred until free cells could be observed in the solution (a cloudy appearance). Residual cellular clumps of vascular bundles and unseparated cells were removed by passing through a 1 mm mesh sieve into a 50 ml falcon tube. These clumps typically represented 10 to 20% of the volume of chopped apple pieces. The separated cells were allowed to settle for an hour, after which excess supernatant was removed leaving an equal amount of liquid as settled cells. At this point the cell homogenate could be stored at 4°C before imaging. Utilising a chemical rather than an enzymic treatment allowed the use of boiling to speed up cell separation, greatly reducing the time used in other tissue maceration methods [1,7,23,24].

Prior to microscopic imaging, harvested cell preparations were re-suspended by gentle flicking of the tube, 30 ul aliquots of suspended tissue were spotted onto clean glass microscopy slides and viewed at 4× under bright field with contrast maximised. Images were collected in a grid like manner to reduce biased selection of cells, using a CoolSnap digital camera and captured using RSImage software, version 1.9.2 (Roper Scientific Ltd, Tucson, Arizona). Images were saved as 24 bit Tiff files.

### Analysis of cell size of different apple cultivars

The size of cells was analysed using the public domain ImageJ software package . For each image an unadjusted image was kept open to check that measurement of cells were consistent with the raw image. The threshold was set so the outline of each cell was clearly differentiated from the background, and then each image was converted to binary (black and white). Each cell was then filled using "Fill holes". Where cells had not been completely separated then the "Watershed" separation was used to separate out the single cells from the clumps. Occasionally intact cells were not filled completely due to small gaps in the outline. If this occurred then the gaps were manually filled and before proceeding with the "Fill holes" again. Areas were calculated using "analyse particles" with a particle size cut-off threshold of 1100 pixels. A skeletonised image was obtained and visually checked that the cells analysed were whole and single (Figure 1B–D).

**Figure 2:**
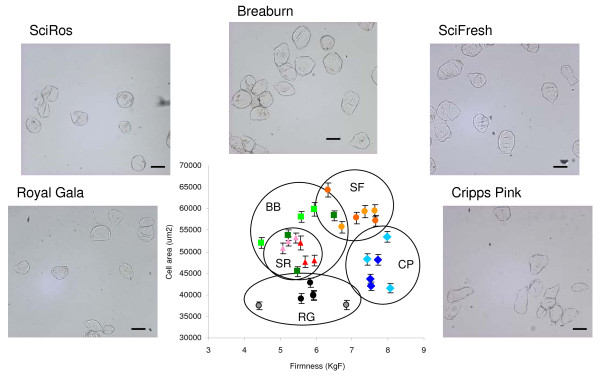
**Comparison of isolated cells from different apple cultivars**. Average size of cells compared to adjacent penetrometer readings. Light tones represent tissue from sun side, darker tones represent shade tissue. Black – (RG) 'Royal Gala', blue – (CP) 'Cripps Pink/Pink Lady™', red – (SR) 'SciFresh/Pacific Rose™', green – (BB) 'Braeburn', orange – (SF) 'Scifresh/Jazz™'. A Representative image of isolated cells from each of the cultivars is presented. Bars represent 200 μM.

It has previously been shown that in some cultivars the blush side (sun exposed) of apples are often firmer than the non blush side (shaded) [26]. To assess the differences between sun and shade sides of fruit and between fruit cultivars, the cell size of 5 common *Malus × Domestica *Borkh. (apple) cultivars with different textures were measured. Three similarly sized apples from standard industry cold stored conditions of 'Braeburn', 'Cripps Pink/Pink Lady'™, 'Scifresh/Jazz'™, 'SciRos/Pacific Rose'™ and 'Royal Gala' cultivars were measured for firmness on the sun and shade side using a penetrometer. For all apple cultivars there was no statistically significant difference in firmness between the sun and shade side (Table 1). There was however, a statistically significant difference in firmness between cultivars, with 'SciFresh' and 'Pink Lady' being firmer than 'Royal Gala' and 'SciRos' (Table 1). Individual apple cells were isolated from each variety (Figure 2). For each apple cells were isolated from cortex tissue adjacent to the penetrometer wound site and approximately 170 individual cells were measured (Table 1, Figure 2). There was no correlation between cell size and firmness either within a cultivar or between cultivars. However it was noted that the firmer apples ('SciFresh' and 'Cripps Pink') had more angular cells compared to the softer cultivars 'SciRos' and 'Royal Gala' (Figure 2).

**Table 1:** Size of apple cells from different cultivars

**Apple cultivar**	**Average weight (g)**	**Sample location**	**Firmness** **(Kgf)**	**± SE**	**Average size (um^2^)**	**± SE**
Royal Gala	168 ± 16	Sun side	5.72	0.87	38280	583
		Shade side	5.78	0.12	40633	636
		**Total Apple**	**5.75**	**0.42**	**39465**	**433**

Pacific Rose	180 ± 7	Sun side	5.24	0.13	52170	696
		Shade side	5.73	0.13	49270	693
		**Total Apple**	**5.49**	**0.13**	**50761**	**408**

Braeburn	198 ± 2	Sun side	5.33	0.53	56606	796
		Shade side	5.74	0.47	52436	718
		**Total Apple**	**5.54**	**0.45**	**54521**	**540**

Jazz	184 ± 8	Sun side	7.23	0.33	58206	841
		Shade side	7.04	0.47	59878	826
		**Total Apple**	**7.13**	**0.40**	**59051**	**589**

Pink Lady	191 ± 4	Sun side	7.82	0.24	47817	731
		Shade side	7.58	0.07	44633	655
		**Total Apple**	**7.70**	**0.08**	**46242**	**494**

**Figure 3:**
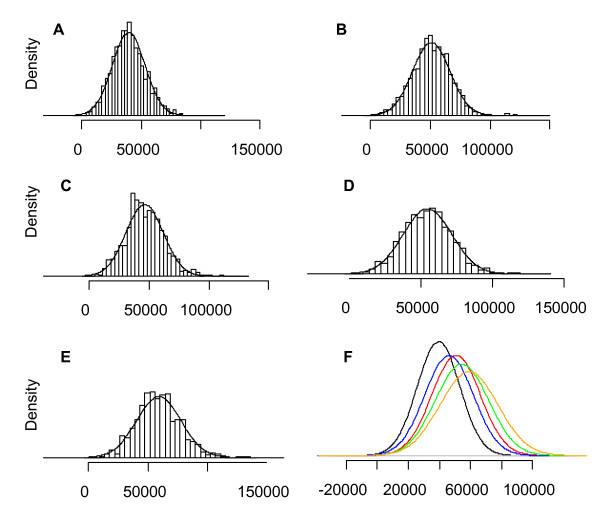
**Histograms of combined cell size data (μM) from different cultivars minimum of 1000 cells**. For each a normal distribution calculated on mean and standard deviation is overlaid. 'Royal gala' (A), 'SciRos/Pacific Rose™' (B), 'Cripps Pink/Pink Lady™' (C), 'SciFresh/Jazz™' (D), 'Braeburn' (E). Comparisons of each of the normal distributions from each of the cultivars (black- 'Royal Gala', blue- 'Pink Lady™', red- 'Pacific Rose™', green-'Braeburn', orange- 'Jazz™' (F).

To further investigate differences in cell size between cultivars, size measurements from each cultivar were combined and histograms of the distributions were plotted Figure 3. Using "rnorm" in the statistical software package "R", a normal distribution of cell sizes was calculated with the mean and standard deviation of sizes from each cultivar using a random generator of 10,000 points. This modelled distribution was plotted on the actual cell distribution and it was found that for all species a normal distribution of cell size occurred (Figure 3A–E). This implies that apples have only one population of cell types in the middle of the cortex tissue. The modelled normal distributions for each cultivar were compared and it was found that each apple cultivar showed a distinct distribution of cell sizes (Figure 3F). The range of cell sizes observed for these cultivars were consistent with previous studies (e.g[19]) which have shown cells with a diameter in the range of 250 uM, assuming a circular area would produce approx 50,000 uM^2 ^(Table 1).

### Isolated Cells are representative of cells from untreated tissue

**Figure 4:**
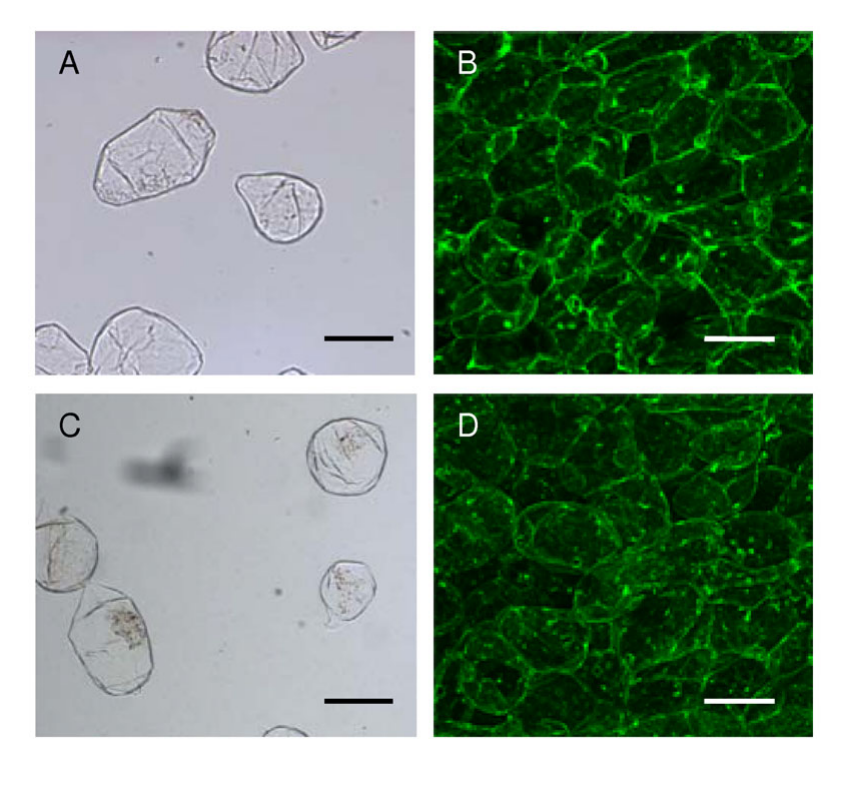
**Comparison of cellular morphology from two cultivars of apple flesh tissue, single cell extraction (A, C) and confocal microscopy of whole section (B, D)**. (A, B) 'Scifresh/Jazz™') apples with more angular cells. (C, D) 'SciRos/Pacific Rose™') apples with rounder cells. Bars represent 200 μM.

When separating cells there is a possibility that cell size and shape can be altered either mechanically or osmotically. To investigate the difference in shapes observed in cells extracted from 'SciFresh' (more angular cells) and 'SciRos' (more rounded cells), these cultivars were chosen for confocal microscopic analysis. Blocks of tissue were taken from the equatorial regions of firm apples of 'SciFresh' and 'SciRos' within 3 weeks of harvest. Each piece comprised a 5–6 mm thick cross-section extending from 5 mm to 15 mm from the fruit surface. Tissue was fixed in 20 g L^-1 ^fresh formaldehyde, 25 g L^-1 ^glutaraldehyde in 0.1 M sodium phosphate buffer pH7.2. A light vacuum was applied to remove as much air as possible from the tissue which was then stored at 4°C in the fixative. Samples for confocal microscopy were washed in 0.1 M sodium phosphate buffer for 2–3 h (3 changes of buffer) and were then sectioned at 800 μm using a Vibratome 1000 (Technical Products International, St Louis Mo) and stained with 0.001 g L^-1 ^acroflavin in 0.1 M buffer for 15 min, washed 3 times in phosphate buffer and mounted on slides with 800–900 μm deep chambers to prevent the coverslip compressing the tissue. Sections were viewed using an Olympus FV1000 confocal microscope (Olympus Corporation, Tokyo, Japan). For each area imaged a stack of approximately 50 individual images was taken at 6 μM intervals. These stacks were used to produce single z projection images. From these confocal images it is clear that the rounded and angularity morphologies seen in the 'SciRos' and 'SciFresh' isolated cells are consistent with the un-separated tissue (Figure 4B, D). To identify any size changes that might have occurred during cell separation, the average longest dimension of the 'SciRos' cells from the confocal tissue section, and 'SciRos' isolated cells were measured. Cells in the confocal sections averaged 268.08 μM (± 50.3), compared to isolated cells that averaged 276.47 μM (± 46.55), demonstrating there was no significant change in size during extraction.

### Cell separation is achieved in other fleshy fruit

**Figure 5:**
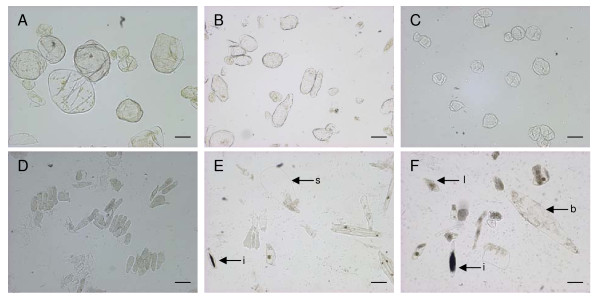
**Cell extractions from other fleshy fruit**. Tomato flesh cells (A), Rock melon flesh cells (B), Immature 'Royal Gala' apple cells (C), Kiwifruit cells (D-F) (D cells from core tissue, E cells from inner pericarp tissue and F cells from outer pericarp tissue). Kiwifruit cells have been marked showing (i) idioblasts, (s) sac cells, (b) big cells and (l) little cells. Bars represent 200 μM.

To test whether this method could be used in other plant tissue, we assessed fleshy tissue from unripe fruit purchased from the local supermarket, including green mature/breaker stage tomato (*Solanum lycopersicum*) (Outer pericarp including skin) (Figure 5A), ripe rock melon (*Cucumis melo CV cantalupensis*) (rind extending into flesh tissue) (figure 5B), and mature unripe kiwifruit (*Actinidea deliciosa*) (outer pericarp, inner pericarp and core tissue extracted separately) (figure 5D–F). Additionally, cortex tissue from immature apple fruit was also tested (approximately 80 DAFB) (figure 5C). It was found that all these showed a good cell separation of flesh tissue: the tomato skin and the rind tissue of the water melon did not separate into individual cells. However, the core tissue from kiwifruit broke down rapidly into individual cells. The immature apples also showed rapid cell disassociation, suggesting cellular linkages are less well formed in rapidly growing fruit. Unlike the apple cells which are fairly homogeneous in morphology, kiwifruit have been previously documented as having a range of cell types in the fruit [27], with the outer pericarp having large cells and small cells, the inner pericarp having long thin cells and idioblasts (cells containing crystalline oxylate) and the core having regular smaller cells [27]. All these classes of cells were observed (Figure 5) suggesting that this method did not exclude certain cell types.

## Conclusion

Here we have shown a robust simple method of isolating single cells from fleshy fruit. Once single cells have been isolated we used a freeware software to measure cell size. In this study we found no correlation between cell size and firmness either within a cultivar, or across cultivars. Interestingly cultivars with more angular cells ('SciFresh' and 'Cripps Pink') have a firmer flesh. From the confocal microscopy images the 'Scifresh' flesh appears to have a higher cell density and therefore greater cell-to-cell contact compared to the 'SciRos' apples. This is consistent with previous findings with firm fleshed 'Granny Smith' apples having more densely packed cells compared to the rounder cells of softer fleshed 'Rubinette' apples [28]. Whether other textural traits, such as juiciness, that has previously been associated with large cells [9] can be linked to cell size in these cultivars is yet to be established. The method presented here would greatly facilitate such comparisons, and allow greater numbers of fruit to be analysed to understand the impacts of orchard and storage factors on fruit morphology.

## Competing interests

The authors declare that they have no competing interests.

## Authors' contributions

PAM carried out the lab work, ICH Helped with the microscopy and image analysis, JWJ and RJS conceived of the project and oversaw the research. All authors read and approved the final manuscript.
